# DNA Helicase–Polymerase Coupling in Bacteriophage DNA Replication

**DOI:** 10.3390/v13091739

**Published:** 2021-08-31

**Authors:** Chen-Yu Lo, Yang Gao

**Affiliations:** Department of BioSciences, Rice University, Houston, TX 77005, USA; cl111@rice.edu

**Keywords:** DNA replication, helicase, polymerase, bacteriophage T7, bacteriophage T4, bacteriophage Φ29

## Abstract

Bacteriophages have long been model systems to study the molecular mechanisms of DNA replication. During DNA replication, a DNA helicase and a DNA polymerase cooperatively unwind the parental DNA. By surveying recent data from three bacteriophage replication systems, we summarized the mechanistic basis of DNA replication by helicases and polymerases. Kinetic data have suggested that a polymerase or a helicase alone is a passive motor that is sensitive to the base-pairing energy of the DNA. When coupled together, the helicase–polymerase complex is able to unwind DNA actively. In bacteriophage T7, helicase and polymerase reside right at the replication fork where the parental DNA is separated into two daughter strands. The two motors pull the two daughter strands to opposite directions, while the polymerase provides a separation pin to split the fork. Although independently evolved and containing different replisome components, bacteriophage T4 replisome shares mechanistic features of Hel–Pol coupling that are similar to T7. Interestingly, in bacteriophages with a limited size of genome like Φ29, DNA polymerase itself can form a tunnel-like structure, which encircles the DNA template strand and facilitates strand displacement synthesis in the absence of a helicase. Studies on bacteriophage replication provide implications for the more complicated replication systems in bacteria, archaeal, and eukaryotic systems, as well as the RNA genome replication in RNA viruses.

## 1. Introduction

Bacteriophages (phage), the lytic viruses that infect bacteria, are the most copious organisms in the environment. The majority of the currently sequenced phages use double-stranded DNA as their genetic materials [1,2], the same as those used in bacteria, archaeal, and eukaryotic cells. Owing to their simplicity, phages have been used as model systems to investigate the fundamental principles of molecular biology. Research on phages by Hershey and Chase seven decades ago established DNA as the genetic material [3]. In recent times, biochemical, biophysical, and structural studies on phage DNA replication have continued to provide mechanistic insights into how DNA is replicated by concerted actions of replicative enzymes [4,5,6,7]. In addition, many enzymes involved in phage DNA replication are utilized in biotechnology applications [8]. A deeper mechanistic understanding of the phage enzymes that are involved in DNA replication is essential for both basic and applied research.

Double-stranded (ds) DNA forms an antiparallel helical structure with the nucleoside base-pairs buried within the center. The dsDNA must be opened up during DNA replication, which is usually catalyzed by the ATPase-coupled motor proteins called DNA helicases (Hel) [9]. DNA polymerases (Pol) perform DNA-dependent DNA synthesis in the 5′-to-3′ direction [10]. Only one strand of the parental dsDNA can be copied continuously, namely the leading strand, while the other lagging strand is synthesized discontinuously in short Okazaki fragments [11]. Being the core components of a multiple protein complex named replisome, Hel and Pol perform the dsDNA unwinding and the leading-strand DNA synthesis cooperatively, and, in conjunction, they set the pace for replisome progression [4,6,7]. In eukaryotic cells, several hundred proteins are tethered to the moving replisome to ensure proper replication of the genome [12,13]. Phages have limited genomes ranging from 10 to 500 kilobases and often only encode tens of genes for their life cycle [5]. Remarkably, phage replisomes with just a handful of components can achieve efficiencies comparable to those of much more complicated replisomes [4,6]. This review surveys replisomes from three diverse phages and discusses the mechanistic basis of Hel–Pol coupling in their DNA replication.

## 2. Hel–Pol Coupling in Bacteriophage T7 DNA Replication

Phage T7 contains a 40-kilobases long linear dsDNA genome that encodes 50 proteins [5]. Replication initiates at a primary origin site located 15% from the left end following RNA transcription [14]. Bidirectional replication is established after replication initiation with concurrent continuous leading-strand and discontinuous lagging-strand DNA synthesis in both directions [14]. Concatemers are involved in subsequent cycles of T7 DNA replication and replication termination [15]. Only three phage proteins, i.e., the gp4, the gp5, and the gp2.5, are needed along with the host factor, thioredoxin (trx), to constitute T7′s simple replisome that performs replication elongation [4]. Gp5 is an 80 kDa protein that belongs to the A family of DNA Pols and has Pol activity for DNA synthesis and exonuclease activity for proofreading [16]. Trx works as a cofactor of gp5, which binds to an insertion loop in gp5 to stabilize the primer-template dsDNA during DNA synthesis [16]. Trx binding stimulates gp5 activity and increases its processivity 80-fold [17,18]. Trx is a redox protein, but the trx stimulation of gp5 is independent of its redox activity [19]. Gp5-trx (the T7 Pol holoenzyme) is responsible for both the leading-strand and the lagging-strand DNA synthesis in T7 [4,17]. The hexameric gp4 has primase activity on its N-terminus to initialize Okazaki fragment synthesis, and Hel activity on its C-terminus to unwind the dsDNA. The Hel domain on gp4 is a member of the superfamily 4 (SF4) Hels and uses the energy from ATP or dTTP hydrolysis to migrate in the 5′-to-3′ direction on the lagging strand DNA [20,21]. The tight DNA binding by gp4 helicase domains promotes substrate engagement of the primase [20]. On the other hand, the primer synthesis by primase domains does not affect helicase translocation [22]. Gp2.5 is a single-stranded (ss) DNA binding protein that protects ssDNA and helps to coordinate the leading- and lagging-strand DNA synthesis [23]. Extensive biochemical, biophysical, and structural biological studies have established how T7 Hel and the leading-strand Pol work cooperatively [21,24,25,26]. Therefore, we use the T7 replisome to discuss the general principles involved in Hel–Pol coupling.

**Figure 1:**
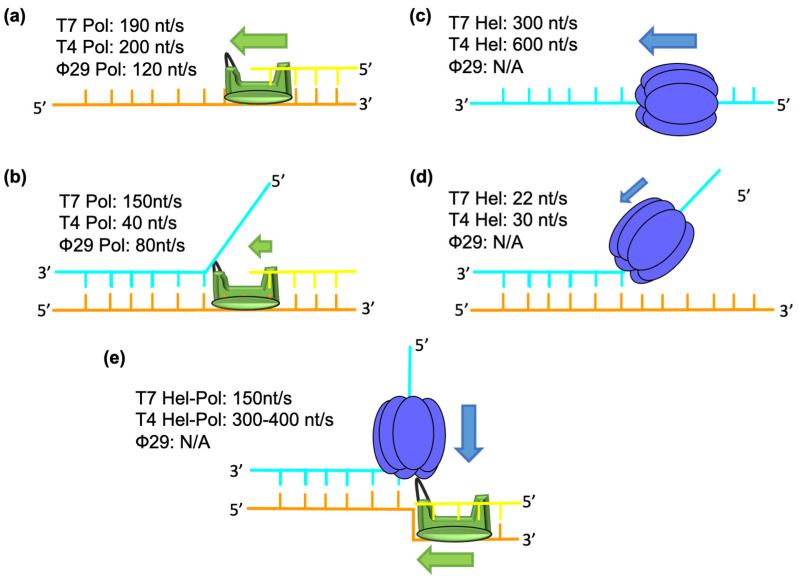
Comparison of the kinetic parameters of dsDNA unwinding in T7, T4, and Φ29 systems. (**a**) The rate of Pols (green) translocation on the ssDNA. (**b**) The rate of Pols (green) translocation on the dsDNA. (**c**) The rate of the hexameric Hels (blue) translocation on the ssDNA. (**d**) The rate of the Hels (blue) translocation on the dsDNA. (**e**) The rate of the coupled Hels (blue)–Pols (green) action on the dsDNA. The arrows indicate the direction of Hel and Pol translocation, with their lengths correlated with the translocation processivity and their thickness correlated with the translocation speed.

T7 Hel and Pol work synergistically to unwind the parental DNA. Comparisons of the DNA unwinding and synthesis kinetics by individual Hel and Pol with the Pol–Hel complex revealed the key barrier for DNA replication. T7 pol contains exonuclease activity which may cause polymerase backtracking when encountering DNA secondary structures [27], so exonuclease-free (exo^−^) T7 pol is often used for kinetic measurements. When acting on a ssDNA template, exo^−^ T7 Pol has a catalytic rate of ~200 nucleotides per second (nt/s) and can synthesize ~800 nucleotides (nt) in a single binding event (Figure 1a) [17]. Although operating at a nearly normal speed of 150 nt/s, exo^−^ T7 Pol can only synthesize 5–6 nt before falling off when it works on a dsDNA substrate (Figure 1b). Moreover, exo^−^ T7 Pol dNTP binding changes from 10–20 μM on a ssDNA template to 40 μM to 270 μM on a dsDNA template, and the dNTP binding constant is negatively correlated with the base-pairing energy [24]. T7 Hel can translocate along ssDNA at rates of up to ~300 nt/s (Figure 1c) [28]. In contrast, T7 Hel has a lower translocation rate of 22 nt/s when unwinding dsDNA (Figure 1d) [24]. Similar to T7 Pol, the unwinding by T7 Hel is sensitive to the base composition of the dsDNA and slows down at high guanine and cytosine (GC) content base-pairs [29]. When working together, T7 Hel and Pol powerfully stimulate each other’s activities [24,26]. T7 Hel enhances dNTP binding and processivity of T7 Pol, while T7 Pol increases the unwinding rate of T7 Hel. T7 Hel and Pol together can catalyze the strand-displacement synthesis at a rate of ~150 nt/s and with a processivity of up to 17 kilobases (Figure 1e) [30,31]. Furthermore, the coupled Hel–Pol is insensitive to the base composition of the dsDNA [24]. In addition to their kinetic coupling during base-pair separation, T7 Hel and Pol are known to interact with each other physically [32,33,34]. The C-terminal tails of T7 Hel are highly negatively charged, and these tails can interact with several positively charged patches on the T7 Pol surface.

**Figure 2:**
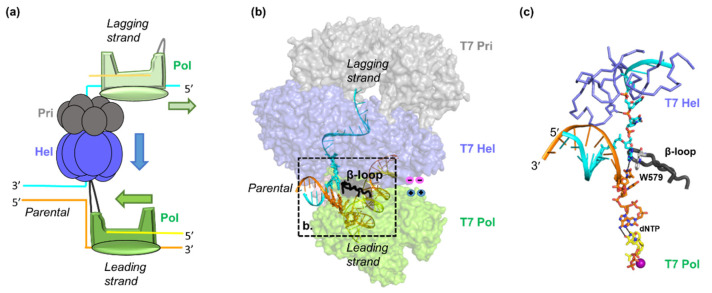
Structure of the T7 Hel–Pol complex on a replication fork. (**a**) Diagram of the replication fork with the coordinated leading-strand and lagging-strand DNA synthesis. (**b**) The Hel–Pol–DNA fork structure. (**c**) A zoom-in view of the DNA fork bound by the T7 Pol and the Hel. The Pol, Hel, and primase are colored green, blue, and grey, respectively. The β-loop for the T7 Pol is shown as black cartoons and resides at the fork junction. The W579 from the β-loop stacks with the first base pair of the parental DNA. The T7 Hel DNA binding loops are shown in a blue ribbon in panel (**c**). The position of the charged–charge interactions between Hel and Pol are indicated by pink and blue symbols. Structures are adapted from [21].

**Figure 3:**
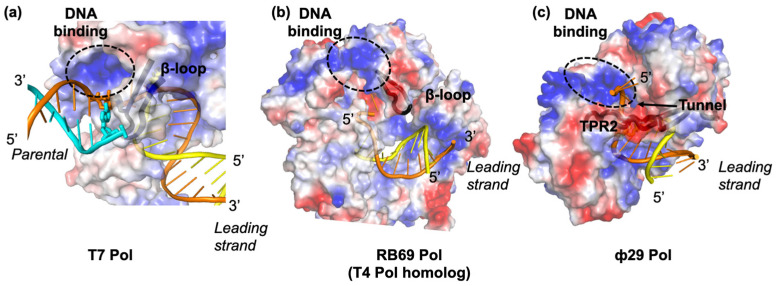
DNA binding in T7 (**a**), RB69 (**b**), and Φ29 (**c**) Pols. The electrostatic surfaces of proteins are shown, with negatively and positively charged surfaces colored as red and blue, respectively. The dsDNA binding surface in T7 Pol and the potential dsDNA binding surfaces in T4 and Φ29 Pols are highlighted with black circles. The β-loops in the T7 Pol and the T4 Pol and the TPR2 domain in the Φ29 Pol are shown as black cartoons. An arrow indicates the TPR2 tunnel in the Φ29 Pol. Structures are adapted from [21,38,39].

Hel and Pol are both motor proteins that can translocate along their DNA substrates. dNTP binding induces the closure of the T7 Pol active site by a finger domain [16,35]. Pyrophosphate release following dNMP addition drives the opening of the finger domain and the translocation of DNA by one nucleotide. T7 Hel forms a lock-washer-shaped hexamer with ATP/dTTP hydrolysis sites at each of the subunit interfaces, and ssDNA binding to its central channel [21]. Sequential hydrolysis at the 5′-DNA end subunit enables subsequent subunit translocation traveling from the 5′-DNA end of the lock-washer to the 3′-DNA end over a distance of 20 Å [21,36,37]. The dNTP incorporation by T7 Pol and the nucleotide hydrolysis by T7 Hel are highly coupled during concerted DNA replication [25]. Recent structures of the T7 Hel–Pol complex on a DNA fork substrate illustrated the structural basis of coupled T7 Hel–Pol translocation (Figure 2a,b) [21]. In the T7 Hel–Pol structure, the parental dsDNA sits between Pol and hexameric Hel. Two daughter strands run in the opposite directions, one entering the T7 Pol site and the other going to the T7 Hel DNA-binding site. The DNA template base for the nascent DNA synthesis is 1 nt away from the fork junction, whereas the DNA in the T7 Hel DNA-binding channel is right next to the fork junction (Figure 2c). The close distances of T7 Hel and Pol to the fork junction agree with the results from DNA protection assays and could be the key for Hel–Pol coupling [25]. A β-hairpin loop (β-loop) on T7 Pol sits at the fork opening with a bulky sidechain W579 stacking against the first base pair in the parental dsDNA. The β-loop and the W579 may facilitate strand separation, reminiscent of the separation pins in monomeric Hels [36]. T7 Pol is located on the C-terminal side of T7 Hel, positioning Hel C-tails adjacent to the positively charged patches on T7 Pol. The electrostatic interactions between T7 Hel and Pol appear to be highly dynamic [34]. There are six negatively charged C-terminal tails on the T7 Hel hexamer and three positive patches on one T7 Pol. The orientation between the parental DNA and the leading-strand Pol is fixed (Figure 3a), whereas the ss-dsDNA junction on the lagging-strand DNA is flexible. Several different interaction modes have been observed in cryo-EM assemblies with only one or two of the T7 Hel C-tails interacting with the T7 Pol in each configuration [21].

Structural and biochemical data of the T7 replisome illustrated the fundamental principles of Hel–Pol coupling. Based on kinetics data, base separation of the parental dsDNA is the major hurdle for Hel or Pol progression [21,24]. In the T7 replisome, Hel and Pol are located right at the fork junction to melt the dsDNA, with each motor pulling one daughter strand into opposite directions. In addition, the β-loop with the W579 sidechain in T7 Pol is located at the fork junction to further facilitate base-pair separation. Many monomeric Hels contain an ATPase domain and a separation pin in the same polypeptide chain and can unwind dsDNA actively regardless of its base-pairing energy [40,41]. However, T7 Hel is sensitive to the base-pairing energy of the dsDNA, suggesting a passive unwinding mechanism [29,42]. T7 Hel becomes an active motor in the presence of T7 Pol, which holds the excluded strand and provides a separation pin to split the dsDNA [16]. Direct interactions between T7 Hel and Pol keep the two motors in the proximal position to unwind dsDNA [21]. T7 Hel and Pol act on two sides of the parental dsDNA, which has a diameter of 20 Å. The segregation and the dynamic Hel–Pol interaction allow large-scale conformational changes in Hel and Pol without sterically crashing into each other [21]. When the protein–protein interactions between Hel and Pol are disrupted by removing the C-terminal tail of gp4, the replication processivity is reduced threefold, but the replication speed is only marginally affected [34]. Hel–Pol interaction also helps the replisome to overcome roadblocks during DNA replication [43,44]. It has been shown that in the absence of a fork DNA substrate, T7 Pol can reside on the side or on the N-terminus of the T7 Hel lock-washer [21,45,46]. The multiple interaction modes of T7 Hel and Pol may help to maintain replisome integrity during transient replication stalling [43].

## 3. Hel–Pol Coupling in Bacteriophage T4 DNA Replication

The phage T4 includes a large circular dsDNA genome of 170 kilobases, which encodes 289 proteins [5]. Four origins have been mapped onto the T4 genome, and replication initiates at one of these origins following RNA transcription [47]. Similar to T7, replication elongation is bidirectional and involves simultaneous leading-strand and lagging-strand DNA synthesis [6]. Origin-dependent replication is inactivated during the development, and later the replisome uses the 3′- end of the lagging-strand for recombination-mediated replication [47]. The reconstitution of the T4 DNA replication elongation complex in vitro requires eight proteins: the gp43 DNA Pol, the gp45 clamp, the gp44/gp62 clamp loader, the gp41 Hel, the gp59 Hel loader, the gp61 primase, and the gp32 ssDNA-binding protein [48,49]. A holo-replicase complex (T4 Pol) comprises gp43 and gp45. The gp45 clamp stabilizes gp43 on the DNA substrate for processive DNA synthesis, and the gp44/62 clamp loader catalyzes gp45′s loading [50]. Gp41 Hel is an SF4 family Hel that hydrolyzes GTP or ATP to unwind the dsDNA along the 5′- to -3′ direction [51]. Gp61 primase physically interacts with the gp41 Hel and regulates its unwinding [52]. Gp59 Hel loader promotes the gp41 Hel loading onto its DNA substrate [53]. Although there are few structural studies in the T4 system, extensive biochemical and single-molecule biophysical studies unveiled how Hel and Pol work cooperatively during DNA replication.

In the T4 replisome, the coupling of the Hel and the Pol drives the leading-strand DNA synthesis. Exo^−^ T4 Pol migrates on the ssDNA at a rate of 200 nt/s (Figure 1a) [48]. When it works on a dsDNA, exo^−^ T4 Pol shows a low strand displacement rate of ~40 nt/s (Figure 1b) [48,54,55]. T4 Hel translocates on the ssDNA at a rate of 600 nt/s, but it can only unwind the dsDNA at a rate of 30 nt/s (Figure 1c,d) [56]. After functional coupling is established between the Hel and the Pol, the T4 replisome shows rapid and processive dsDNA unwinding and the leading-strand DNA synthesis at a rate of 300–400 nt/s (Figure 1e) [54,55]. In addition, the mechanics of the T4 Hel–Pol catalyzed DNA unwinding have been scrutinized using single-molecule optical tweezers [54,56]. Mechanical forces added to the DNA substrates destabilize the dsDNA and enhance the motors’ unwinding efficiency. When a low force is applied, the T4 Pol will stall or regress on the DNA substrate due to primer removal by exonuclease activity of T4 Pol. Similarly, with low force on the dsDNA, the T4 Hel moves six times slower than with high force, and pauses at high GC content base pairs [54,55]. When coupled, the T4 Hel–Pol can synthesize DNA rapidly and processively regardless of the applied force.

The T4 Hel and Pol migrate on two daughter strands of DNA. Considering their cooperativity in unwinding DNA, the two must reside right at the replication fork to prevent replication fork regression and backtracking each other, similar to the T7 replisome (Figure 2a). T4 Hel forms a hexamer and likely performs dsDNA unwinding through a sequential mechanism similar to T7 Hel [57]. T4 Pol is a member of the B family of DNA Pols and shares 62% identity with the phage RB69 Pol, a model protein for studying the structure and mechanism of B-family Pols [58,59]. Previous footprinting results indicated that T4 Pol protects 3–6 nt of an unwound template at the primer-template end, longer than that in T7 Pol [60]. Structures of RB69 Pol suggested that a positively charged surface near the template-exiting site on RB69 Pol can potentially bind the template DNA (Figure 3a,b). A loop near the positively charged surfaces may approach the fork junction to facilitate dsDNA unwinding [38]. Further studies are needed to clarify T4 Pol’s interaction with its DNA substrate. In addition, no physical interaction was found between the T4 Hel and the Pol [61]. Swapping Pols between the T4 and the T7 replisomes maintained coupled unwinding, suggesting that the direct Pol-Hel interaction may not be needed for the functional Hel–Pol coupling [24,26,54]. Alternatively, T4 Hel and T4 Pol may interact indirectly through joint binding partners during DNA replication. For instance, the T4 Hel loader gp59 can interact with both T4 Hel and Pol [62].

## 4. Bacteriophage Φ29 DNA Replication without a Helicase

Bacteriophage Φ29 genome is a 19-kilobases linear dsDNA. Φ29 mainly uses four proteins to fulfill the DNA replication: the terminal protein (TP), the DNA Pol p2, the ssDNA-binding protein p5, and the double-stranded binding protein p6 [63,64,65]. Interestingly, no Hel is found in the Φ29 genome. The p6 forms a nucleoprotein complex at the dsDNA end to help to initiate Φ29 DNA replication. The TP protein binds to the 5’- end of DNA and acts as a protein primer for initializing replication. The P2 is the DNA Pol that performs the DNA synthesis. The p5 protects ssDNA intermediates during replication. Replication of Φ29 genome is thought to start from both ends of the genome with Φ29 Pol performing the strand-displacement DNA synthesis without involving lagging-strand Okazaki fragments [64].

The Φ29 Pol is a 66 kDa B-family DNA Pol [39]. Unlike other Pols, the Φ29 Pol performs efficient and processive strand displacement synthesis without a Hel [66]. The Φ29 Pol translocates at a rate of ~120 nt/s on the ssDNA (Figure 1a) [67,68]. The rate is decreased slightly to 80 nt/s, and the dNTP binding is reduced 2-fold when the Φ29 Pol works on the dsDNA substrate (Figure 1b) [68]. Furthermore, Φ29 Pol pauses frequently at the high GC-content DNA [67]. These results imply that Φ29 Pol is moderately sensitive to the dsDNA base-pairing energy compared to T4 Pol and T7 Pol [24,55]. Φ29 Pol harbors two unique insertion domains: the TP Region 1 (TPR1) binds to the TP and DNA, and the TP Region 2 (TPR2) contributes to Pol processivity and strand-displacement synthesis [39,69]. Specifically, the TPR2, palm, finger, and exonuclease domains form a tunnel surrounding the template DNA (Figure 3c) [39]. The tunnel is about 10Å in width, which can only accommodate the ssDNA binding. The TPR2 tunnel may help to exclude the unwound ssDNA and prevent Pol dissociation or regression during strand displacement synthesis [67]. In addition, positively charged surface regions exist right next to the TRP2 tunnel and may help to hold the parental dsDNA, similar to those in T7 Pol and T4 Pol (Figure 3a–c). Thus, the Φ29 replication system demonstrated that DNA synthesis itself can drive strand separation, albeit with lower efficiency. Interestingly, a recent single-molecule study suggested that the Hel ATP hydrolysis may not be required for parental DNA unwinding in bacterial replisomes [70].

## 5. Conclusions

Studies on T7 and T4 bacteriophages have elucidated the general principles of Hel–Pol coupling in DNA replication. The melting of the parental dsDNA is a critical barrier for DNA replication. Hel and Pol are passive motors on their own but become an active motor when coupled. The T7 Hel and Pol reside right at the replication fork, and each pulls one daughter-strand DNA to separate the dsDNA. In addition, a separation pin from the T7 Pol may facilitate strand separation. Direct protein–protein interaction would help to maintain the replisome integrity during transient uncoupling, but it is not strictly required for the coupled Hel–Pol action. Interestingly, with a unique tunnel that circles the template strand to prevent Pol dissociation and regression, the Φ29 Pol can catalyze dsDNA unwinding in the absence of a Hel motor.

Although many more proteins are involved in bacterial, archaeal, and eukaryotic DNA replication, Hel and Pol form the core of their replisomes [7]. Bacterial and eukaryotic mitochondrial replisomes share similarities with those in phages to the extent that Hels migrate on the lagging strand to couple with the leading-strand DNA synthesis. In contrast, archaeal and eukaryotic Hels are on the leading strand. Hel itself holds the parental DNA with a protein loop stacking at the fork opening [71]. Even though the coupling mechanism between Hel and Pol on the same strand of DNA is not fully understood, the coupled Hel–Pol action is imperative for DNA replication and repair [72]. In addition, RNA viruses also encode their own Hels when the genome sizes permit [73]. The roles of RNA Hel in RNA unwinding and replication require further investigation.

## Data Availability

Not applicable.
